# Sex and age differences in antibody responses to seasonal influenza vaccination are mediated by estrogenic upregulation of NF-κB and TNF signaling in B cells

**DOI:** 10.1128/mbio.01435-26

**Published:** 2026-09-10

**Authors:** Han-Sol Park, Anna Yin, Weiqiang Zhou, Eliane F. E. Wenstedt, Anne Jedlicka, Amanda Dziedzic, Annie Werner, Katherine Z. J. Fenstermacher, Huifen Li, Patrick J. Shea, John S. Lee, Patricia Gearhart, Robert W. Maul, Sjoerd A. A. Van Den Berg, Richard E. Rothman, Hongkai Ji, Sean X. Leng, Andrew Pekosz, Sabra L. Klein

**Affiliations:** 1 W. Harry Feinstone Department of Molecular Microbiology and Immunology, Johns Hopkins Bloomberg School of Public Health 25802 , Baltimore, Maryland, USA; 2 Department of Biostatistics, Johns Hopkins Bloomberg School of Public Health 25802 , Baltimore, Maryland, USA; 3 Department of Clinical Chemistry, Erasmus Medical Center 6993, Rotterdam, the Netherlands; 4 Department of Emergency Medicine, The Johns Hopkins School of Medicine 1500 , Baltimore, Maryland, USA; 5 Division of Geriatric Medicine and Gerontology, Center on Aging and Immune Remodeling, Department of Medicine, Johns Hopkins University School of Medicine 1500, Baltimore, Maryland, USA; 6 Laboratory of Molecular Biology and Immunology, National Institute on Aging, NIH 189361, Baltimore, Maryland, USA; Georgia Institute of Technology, Atlanta, Georgia, USA

**Keywords:** sex steroids, inflammation, neutralizing antibodies, plasmablast, aging, influenza vaccines

## Abstract

Sex differences in the humoral immune responses to the seasonal quadrivalent influenza vaccine (QIV) in young adults (YA; 18–49 years old) or high-dose QIV in old adults (OA; 75+ years old) were analyzed to determine how age-related changes, including in steroids, impact sex differences in B cells. Among YAs, females had greater H3N2, but not H1N1, neutralizing antibody titers, and greater proportions of hemagglutinin (HA)+ CD19+ B cells and HA+ memory B cells than males through 28 days post-vaccination (DPV), that was not observed among OAs. Machine learning algorithms illustrated that baseline (0 DPV) steroids, including 17-hydroxyprogesterone, estrogens, and testosterone, as well as HA+ CD19+ B cells and HA+ antibody-secreting B cells (ASCs), were major predictors of seroconversion at 28 DPV, particularly in YA. Single-cell RNA sequencing demonstrated that CD19+ B cells from YA females had greater transcriptional activity at 7 DPV than YA males, with upregulation of genes with estrogen-response elements (EREs) along NF-κB-mediated TNF signaling pathways in B-cell subsets, which was mitigated in OA. Estradiol treatment of ASCs from YA females, but not males, increased the number and size of HA+ IgG+ cells and expression of ERE genes along the NF-κB-mediated TNF signaling pathway,that was inhibited by an estrogen receptor antagonist. Pharmacological inhibition of either NF-κB or TNF signaling blocked the ability of E2 to upregulate antibody secretion in cells from YA females. This study provides mechanistic insights into estrogen-mediated increases in influenza vaccine-induced antibody responses among reproductive-aged females and suggests a role for estrogen signaling in the reduction of sex differences in vaccine-induced immunity with old age.

**IMPORTANCE:**

Sex differences in influenza vaccine-induced immune responses become less pronounced with old age, which we hypothesize could be related to changes in circulating gonadal steroids. Our study shows that after receipt of the seasonal influenza vaccine, young adult females, who have elevated estrogenic activity, have more B cells that recognize influenza hemagglutinin; their B cells have greater activity along estrogen signaling and inflammatory pathways, and mount stronger antibody responses than young adult males, with these sex differences being mitigated in old adults. We identify estrogen as a key driver of sex differences in influenza immunity by showing that *ex vivo* estradiol increases antibody production by B cells through the estrogen receptor and engagement with NF-κB. These findings help explain the biological basis for sex differences in vaccine immunity and suggest that the hormonal environment, not just chronological age, shapes how well a person responds to vaccination.

## INTRODUCTION

Across the life course, biological sex and age are fundamental modifiers of immunity to infectious diseases and the response to vaccination. Females tend to mount stronger immune responses than males (1–3), but these sex differences in immunity are not constant and change with age. There is an intersection between sex and age, whereby the impact of aging on the immune system differs in males and females (4, 5). The implications of the interaction between sex and age are clearly demonstrated by the epidemiology and clinical manifestations of respiratory viral diseases, such as influenza and COVID-19 (6, 7). Influenza A viruses (IAVs) have caused four of the five pandemics during the past century, with seasonal influenza epidemics resulting in approximately 650,000 deaths worldwide, with people 65+ years of age being at a higher risk of severe outcomes (8).

Of the few human influenza vaccine trials that have considered sex differences in vaccine outcomes, in adults of reproductive ages (18–49 years), females have greater antibody titers (i.e., either neutralizing or hemagglutination inhibition [HAI] titers) than males following receipt of the seasonal influenza vaccine (9–12). The sex difference in antibody responses is influenced by both age and pre-existing immunity to selected IAV and influenza B virus (IBV) strains (11, 13). During the 2008–2009 influenza season, adult females, for example, produced more neutralizing antibodies against the H3N2 and IBV vaccine virus strains, but not against the H1N1 vaccine virus strain (11). During the 2017–2018 influenza season, adult females mounted more robust neutralizing responses to H3N2 vaccine virus compared to males (14). A recent meta-analysis of 19 randomized controlled trials of influenza vaccination revealed a slightly higher rate of seroconversion in females compared to males, with the sex differential effect being greatest among individuals <65 years of age (15).

Among individuals aged 65+ years, antibody titers to both the standard and high-dose seasonal influenza vaccine are also greater in females than males (15, 16), with greater season-to-season durability of immunity against influenza among older females than males, particularly in response to H3N2 and IBV (13). Both younger age and greater concentrations of circulating estradiol (E2) are associated with greater monovalent 2009 H1N1 vaccine-induced immunity (9), suggesting that declining gonadal steroids, and not just chronological aging, could reduce immunity following vaccination. In one study, estrogen therapy in postmenopausal women increased antibody responses to seasonal influenza vaccines (17). Limited data also suggest that sex differences in influenza-vaccine-induced antibody responses involve the effects of testosterone on lipid biosynthesis in males (11). Missing from the current literature is a deep interrogation into how biological sex, age, and gonadal steroids alter signaling pathways in B cells to cause sex differences in antibody responses following vaccination.

Herein, we conducted parallel studies of young adult (YA) healthcare workers ages 18–49 at the Johns Hopkins Health System (JHHS) receiving the standard dose of the quadrivalent influenza vaccine (QIV) and community-dwelling older adults at least 75 years of age (OA) in Baltimore, Maryland, receiving the recommended high-dose QIV. We show that estrogen impacts signaling through NF-κB and TNF pathways in B cells, resulting in increased vaccine-induced antibody responses in YA females compared to males. In OA, these sex differences are mitigated, in part because of reduced estrogen signaling in B cells from OA females.

## RESULTS

### Patient demographics

During the 2019–2020 influenza season, 82 YA healthcare workers (HCW) at the JHHS between the ages of 18 and 49 years old were enrolled prior to receipt of the required seasonal standard dose inactivated QIV and completed sample collection (Table 1). The YA cohort comprised 43 males and 39 females, with an overall average age of 30 years. The YA participants consisted of 50% White, 17% Black or African American, 17% Asian, 2% American Indian or Alaskan Native, 10% other, and 4% with missing race data. Among the YA females, 26 (68%) were on birth control (BC), none were pregnant, none had a hysterectomy, and none were on menopause hormone therapy (MHT).

**TABLE 1:** Participant demographics and clinical comorbidities for the young adult and old adult cohorts during the 2019–2020 influenza vaccination season^*a*^

Parameter	Young adult cohort, 18–49 years old				Old adult cohort, 75+ years old			
	Male	Female	Total	*P*-value	Male	Female	Total	*P*-value
*n*, (%)	43 (52)	39 (47)	82 (100)		37 (44)	47 (56)	84 (100)	
Age, mean (SD)	29.3 (6.2)	30.9 (7.5)	30.0 (6.9)	0.29	85.5 (5.3)	83.7 (5.1)	84.5 (5.3)	0.13
BMI, mean (SD)	25.8 (3.8)	26.6 (5.4)	26.2 (4.6)	0.42	27 (4)	25.5 (5.1)	26.2 (4.6)	0.08
Race, *n* (%)				**0.02^*b*^**				1.0
White	19 (44)	22 (56)	41 (50)		32 (86)	38 (81)	70 (83)	
Black/African American	4 (9)	10 (26)	14 (17)		2 (5)	3 (6)	5 (6)	
Asian	12 (28)	2 (5)	14 (17)		0 (0)	0 (0)	0 (0)	
American Indian or Alaska Native	2 (5)	0 (0)	2 (2)		0 (0)	0 (0)	0 (0)	
Other	4 (9)	4 (8)	8 (10)		0 (0)	0 (0)	0 (0)	
N/A	2 (5)	1 (3)	3 (4)		3 (8)	6 (13)	9 (11)	
Hispanic/Latino, *n* (%)	8 (19)	7 (18)	15 (18)	1.0	–	–	–	–
Breastfeeding, *n* (%)	–	1 (3)	1 (1)	–	–	–	–	–
Hysterectomy, *n* (%)	–	0 (0)	0 (0)	–	–	7 (15)	7 (8)	–
Immunosuppressive medication, *n* (%)	2 (5)	1 (3)	3 (4)	1.0	0 (0)	0 (0)	0 (0)	
Autoimmune disease, *n* (%)	2 (5)	3 (8)	5 (6)	0.66	20 (54)	36 (77)	56 (67)	**0.03**
Cardiovascular disease, *n* (%)	1 (2)	3 (8)	4 (5)	0.34	8 (22)	7 (15)	15 (18)	0.42
Asthma, *n* (%)	2 (5)	3 (8)	5 (6)	1.0	0 (0)	3 (6)	3 (4)	0.25
Reproductive disease, *n* (%)	0 (0)	4 (10)	4 (5)	**<0.001**	–	–	–	–
Cancer, *n* (%)	0 (0)	1 (3)	1 (1)	0.47	16 (43.2)	17 (36.2)	33 (39.3)	0.65
H3N2 seroprotection at 0 DPV, *n* (%)	11 (28)	22 (63)	33 (45)	**0.003**	14 (44)	16 (37)	30 (40)	0.57
H3N2 seroprotection at 28 DPV, *n* (%)	34 (87)	33 (94)	67 (91)	0.44	22 (69)	32 (74)	54 (72)	0.59
H3N2 seroconversion, *n* (%)				0.27				0.66
Non-responder (<4 FC)	14 (36)	16 (46)	30 (41)		20 (63)	29 (67)	49 (65)	
Responder (>4 FC)	25 (64)	19 (54)	44 (59)		12 (37)	14 (33)	26 (35)	
nAb data missing, *n* (%)	4 (9)	4 (10)	8 (10)		5 (14)	4 (8)	9 (11)	

^*a*^ "–" indicates data that are missing or not applicable and, therefore, where *P*-values/significance cannot be calculated.

^*b*^ Bold terms indicate statistically significant *P* values.

We concurrently enrolled community-dwelling old adults (OAs) in Baltimore above 75 years of age who had not yet received the high-dose seasonal QIV. A total of 84 participants were enrolled and completed sample collection, comprising 37 males and 47 females with an overall average age of 84.5 years (Table 1). The OA cohort was predominantly White (83%, *n* = 70) with 6% (*n* = 5) identifying as Black or African American and 11% (*n* = 9) missing race data. Among the OA females, 8% (*n* = 7) had a previous hysterectomy, and none were on MHT.

### Influenza vaccine compositional changes 2015–2020 season

**Fig 1:**
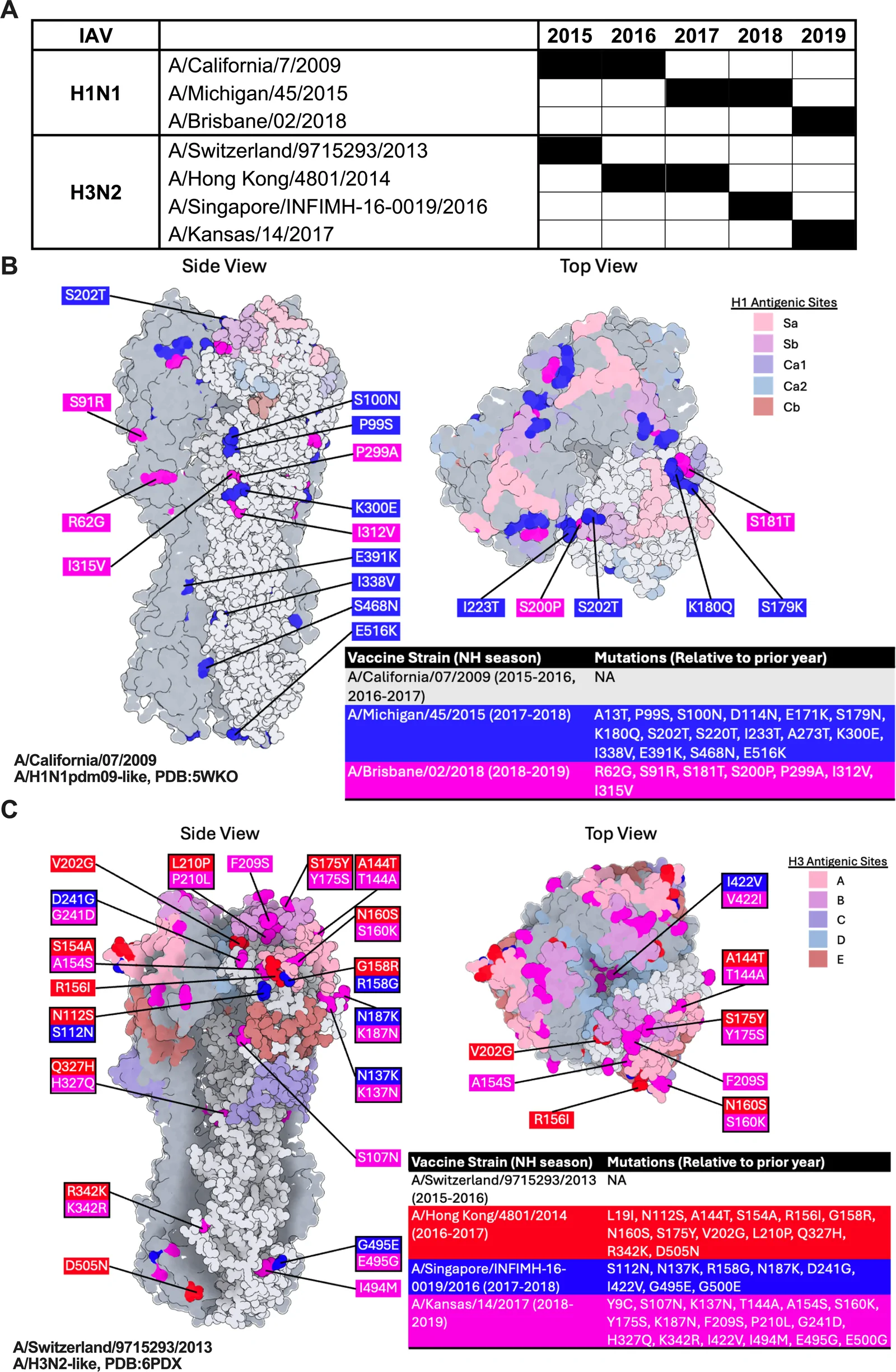
Strain changes in the Northern Hemisphere QIV formulation from 2015 to 2019. (**A**) H1N1 and H3N2 components of the Northern Hemisphere inactivated influenza vaccine from 2015 to 2019. (**B**) HA trimers from A/H1N1 (A/California/07/2009 PDB:5WKO) and (**C**) A/H3N2 (A/Switzerland/9715293/2013 PDB:6PDX) were visualized using Protein Imager. The major antigenic sites are shaded in pastel colors, and amino acid changes in the vaccine strains are indicated with the specific amino acid substitution and the vaccine strain in which it was present. Boxes with two or more amino acid changes indicate multiple changes during this timeframe at that specific site. The H1N1 components collectively had fewer amino acid changes in antigenic sites compared to the H3N2 components.

In 2019, the egg-based QIV composition consisted of A/Brisbane/02/2018(H1N1)pdm09-like virus, A/Kansas/14/2017(H3N2)-like virus, B/Colorado/06/2017-like virus (B/Victoria/2/87 lineage), and B/Phuket/3073/2013-like virus (B/Yamagata/16/88 lineage) in accordance with the World Health Organization’s (WHO) vaccine recommendations for the Northern Hemisphere (18). From 2015 to 2019, the H1N1 vaccine component changed three times, whereas the H3N2 vaccine component changed four times (Fig. 1A). To determine how these changes in the vaccine components affected antigenic sites, the hemagglutinin (HA) trimer structures from A/H1N1 (A/California/07/2009 PDB:5WKO) and A/H3N2 (A/Switzerland/9715293/2013 PDB:6PDX) were visualized with Protein Imager, and amino acid changes between the vaccine components were mapped. The H1 HA protein used in the 2019 influenza vaccine had 23 amino acid changes relative to the vaccine strain used in 2015, with many of them located in or near antigenic sites (Fig. 1B). In contrast, the H3 HA protein had 38 amino acid changes, with 12 of those specific sites having changed twice (e.g., amino acids 187, 175, and 160) during this period (Fig. 1C). These data indicate that individuals vaccinated with the recommended Northern Hemisphere influenza vaccine formulation were exposed to H3 components that had more antigenic site changes than the H1 component, which could impact pre-existing immune responses.

### YA females have greater pre- and post-vaccination neutralizing antibody titers against H3N2 than YA males, with sex differences mitigated in OA

**Fig 2:**
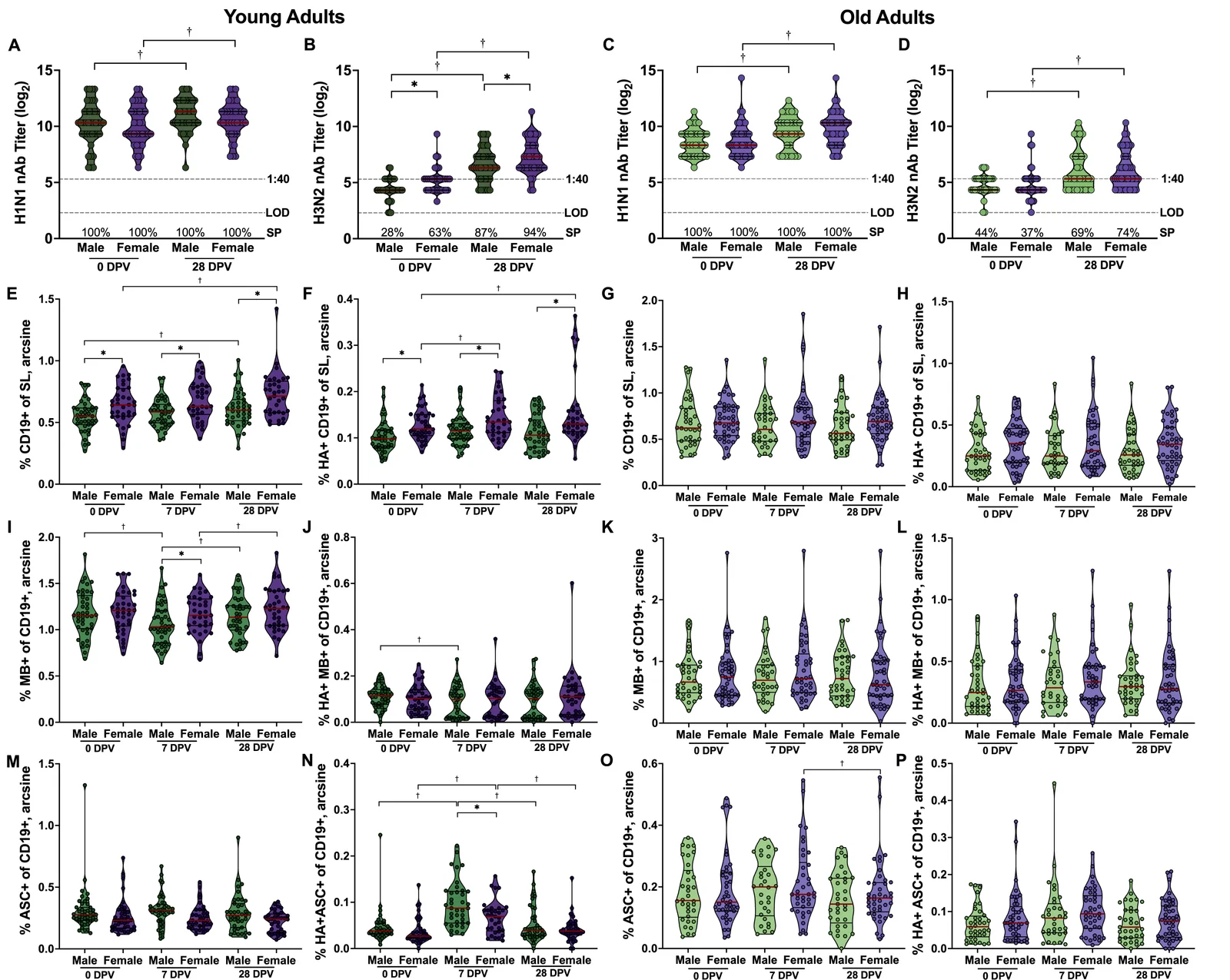
Young adult, but not old adult, females had greater influenza-specific immune responses than males. Among young (**A**) and old (**C**) adults, regardless of sex, H1N1 neutralizing antibody (nAb) titers were above the seroprotection cutoff (nAb titers ≥1:40) at 0- and 28 DPV. In response to H3N2, (**B, D**) 28% of male and 63% of female young adult participants and 44% of male and 37% of female old adult participants had nAb titers that were above the seroprotection cutoff prior to vaccination (0 DPV). Dashed lines represent the limit of detection (LOD) and log_2_ (1:40 titer) for seroprotection. Proportions of total and HA+ CD19+ B cells (**E–H**), memory B cells (MB; **I–L**), and antibody-secreting cells (ASC; **M–P**) were quantified by flow cytometry in young and old adult participants. Total and HA+ CD19+ B-cell populations were gated from single lymphocytes (SL) and MB and ASC populations were gated from CD19+ B cells. Cell population frequencies (%) were arcsine transformed prior to analyses. Data collected across multiple time points were analyzed by linear mixed effects regression models with an interaction term for sex and time point, adjusting for continuous age. Marginal means with 95% confidence intervals are plotted. Young and old adults were analyzed independently unless specified. * represents *P* < 0.05 for sex differences and † represents *P* < 0.05 for increases over time.

Sex differences in neutralizing antibody (nAb) responses to IAVs are typically limited to pandemic strains of H1N1 and seasonal strains of H3N2 (9, 13, 14). Regardless of age group or sex, all participants (100%) had nAb titers against H1N1 influenza that were well above that for seroprotection (≥1:40 titer, log2) prior to vaccination, suggesting strong pre-existing immunity against H1N1 in 2019–2020 (Fig. 2A and C). Despite strong pre-existing nAb titers against H1N1 prior to vaccination, males and females similarly increased nAb titers against H1N1 between 0 days post-vaccination (DPV) and 28 DPV, with no statistically significant sex differences observed, regardless of age. The average fold change in H1N1 nAb titers between 28 and 0 DPV was 1.7 for YA females and 3.1 for YA males (*P* = 0.13). Among OA, the average fold change in H1N1 nAb titers was 2.6 for females and 2.1 for males (*P* = 0.17). These data suggest that although influenza vaccination was able to induce nAb titers against H1N1 for YA and OA, most participants (*n* = 61, 82% of YA and *n* = 51, 68% of OA) did not seroconvert for H1N1 (<4-fold change). Even when YA and OA were separated between non-responders (<4-fold change) and responders (≥4-fold change), sex differences in nAb titers against H1N1 were not observed (Fig. S1A and B).

There were significantly lower pre-existing nAb titers against H3N2 than H1N1 influenza at 0 DPV. YA females had greater H3N2 nAb titers both prior to and 28 DPV than YA males, with no sex differences in H3N2 nAb titers observed among OA (Fig. 2B and D). When YA and OA were separated into non-responders and responders, sex differences in nAb titers were only apparent among YA responders, in which females had greater nAb titers against H3N2 than males (Fig. S1C and D). The average fold change in H3N2 nAb titers between 28 DPV and 0 DPV was 6.6 for males and 8.9 for females among YA (*P* = 0.55). In OA, the average fold change for H3N2 nAb titers was 4.8 for males and 3.9 for females (*P* = 0.59). In response to H3N2, 36% of male (*n* = 14) and 45.7% of female (*n* = 16) YA participants and 62.5% of male (*n* = 20) and 67.4% of female (*n* = 29) OA participants did not seroconvert for H3N2 (<4-fold change). Together, these data suggest that YA female responders were driving the sex differences in nAb responses against H3N2. While our study was not powered to separate out types of birth control (BC), we explored whether BC use could impact nAb titers to either H1N1 or H3N2; we compared YA females who reported using BC with those not using BC. BC use did not impact titers of nAb against either H1N1 (Fig. S1E) or H3N2 (Fig. S1F).

### YA females maintain greater frequencies of HA+ B cells than YA males before and after vaccination, with sex differences mitigated in OA

We used flow cytometry to measure total and HA+ B-cell frequencies in peripheral blood mononuclear cell (PBMC) samples among YAs and OAs collected before and after seasonal influenza vaccination (Fig. S2). Prior to and after vaccination, YA females maintained significantly higher frequencies of total and HA+ CD19+ B cells compared to YA males (Fig. 2E and F), which was driven by responders who seroconverted (Fig. S3A and B). In contrast, OA females and males had similar frequencies of total and HA+ CD19+ B cells, regardless of seroconversion status (Fig. 2G and H; Fig. S3C and D). Total memory B-cell frequencies (defined as CD3e^−^CD14^–^CD16^–^CD19^+^IgD^−^CD71^−^) were greater among YA females than males at 7 DPV (Fig. 2I), which was caused by non-responder females with elevated pre-existing immunity (Fig. S3E). In contrast, HA+ memory B cells were greater in PBMCs from YA females than males at 28 DPV, and this was specific to responders who seroconverted (Fig. 2J; Fig. S3F). In contrast, OA females and males had similar frequencies of total and HA+ memory B cells, regardless of seroconversion status (Fig. 2K and L; Fig. S3G and H). ASCs or plasmablasts (defined as CD3e^−^CD14^–^CD16^–^CD19^+^IgD^−^CD71^+^CD20^−^CD38^+^) are the primary circulating B-cell type that produces antibody, with peak frequencies occurring within 1 week after vaccination (19). Changes in frequencies of total ASCs were not observed over time in either YA or OA males or females (Fig. 2M and O; Fig. S3I and K). In contrast, proportions of HA+ ASCs peaked at 7 DPV in YA (Fig. 2N) but not OA (Fig. 2P), which was driven primarily by YA male responders (Fig. S3J). OA males and females, regardless of seroconversion, did not exhibit a peak in total or HA+ ASCs at 7 DPV (Fig. 2O and P; Fig. S3K and L).

Age-associated B cells (ABCs), also referred to as atypical memory B cells, accumulate in circulation with increasing age and are often associated with immunosenescence (20). Previous studies illustrate that older females tend to have greater numbers of ABCs than age-matched males or younger-aged females and males, particularly in autoimmune disease patients (20). We explored frequencies of ABCs (defined as CD3e^−^CD14^–^CD16^–^CD19^+^CD21^–^CD11c^+^Tbet^+^) (21) in a small subset of YA (*n* = 4 for proof of principle) and all the OA participants and observed that total and HA+ ABCs were only found in high frequencies among OA vaccinees prior to vaccination (Fig. S4A and B). Among OA only, we analyzed whether ABC frequencies changed following vaccination. ABC frequencies did not change in either OA males or females after vaccination and did not differ between the sexes (Fig. S4C and D).

### Concentrations of steroids, precursors, and metabolites differ based on sex and age

**Fig 3:**
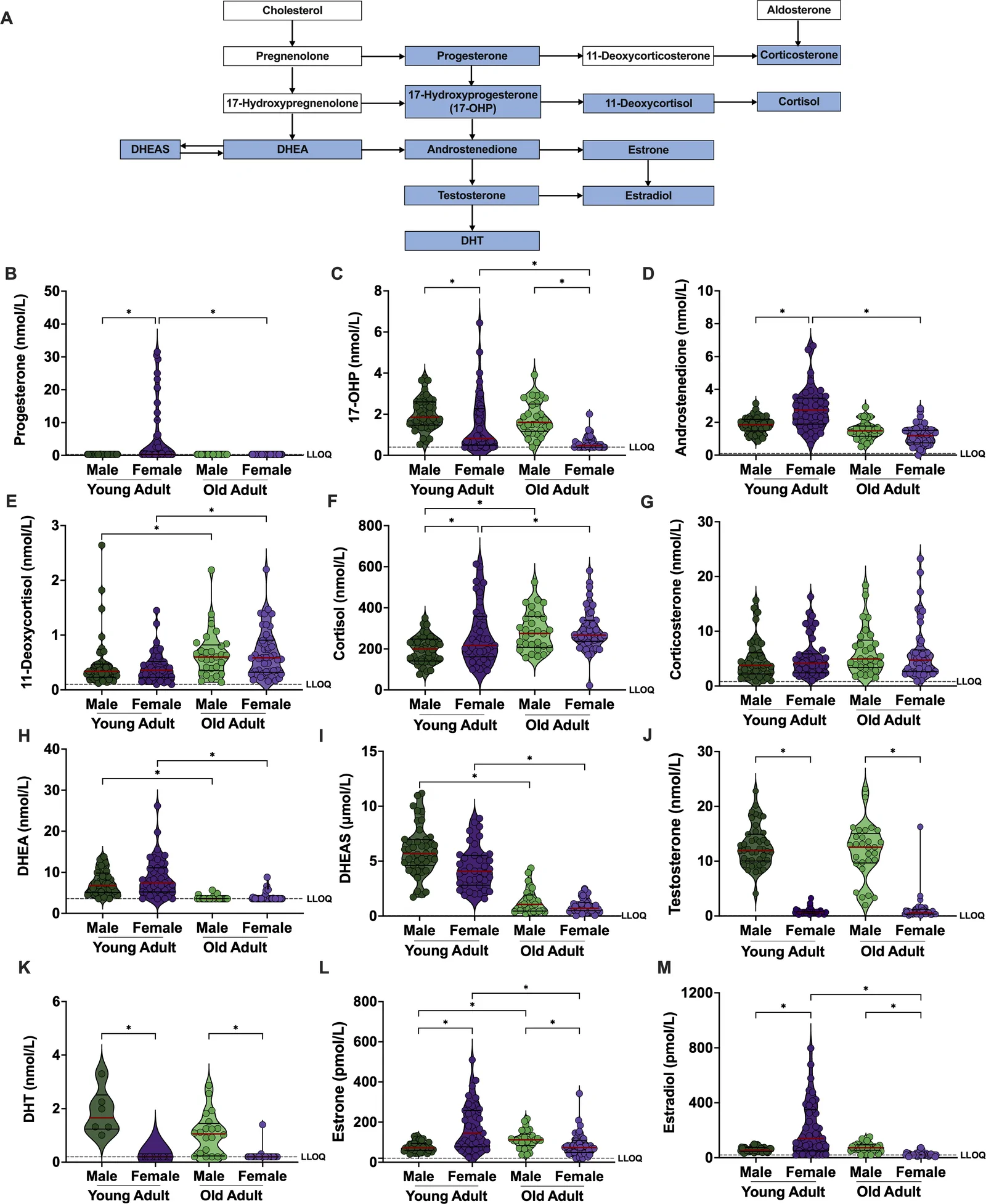
Prior to vaccination, young and old adults had distinct steroid profiles among male and female participants. (**A**) The steroid hormone biosynthesis pathway, with those measured by mass spectrometry highlighted in blue. (**B–M**) Baseline concentrations (mmol/L, nmol/L, or pmol/L) of steroids, as well as their precursors and metabolites, were measured from peripheral blood samples collected prior to vaccination (0 DPV) and compared by sex and age using Kruskal-Wallis with Benjamini-Hochberg post hoc corrections. Dashed lines represent the lower limit of quantification (LLOQ). * represents sex or age differences at *P*adj < 0.05.

We have shown that gonadal steroids correlate with sex and age differences in influenza vaccine-induced immunity in humans and mice (9). In mice, estradiol (E2) causes greater influenza vaccine-induced immunity and protection in both young- and old-aged females by increasing germinal center B-cell immune responses (22). Unlike our previous studies (9, 22), steroids in this study were measured by mass spectrometry in plasma samples collected prior to vaccination, allowing for more accurate assessment of steroid biosynthesis (Fig. 3A).

Progesterone is a precursor for most steroids, and YA females had greater concentrations than either YA males or OA of either sex (Fig. 3B). 17-Hydroxyprogesterone (17-OHP; Fig. 3C) and androstenedione (Fig. 3D) concentrations also were greater in YA females than males, with age-related reductions apparent among females, but not males. Both 11-deoxycortisol (Fig. 3E) and cortisol (Fig. 3F) increased with age in both males and females. Corticosterone was measured and was not different among YA and OA males and females (Fig. 3G).

Dehydroepiandrosterone (DHEA) (Fig. 3H) and dehydroepiandrosterone sulfate (DHEAS; Fig. 3I) are steroid precursors that showed an age-associated decline in both males and females. Androgens, including testosterone (Fig. 3J) and dihydrotestosterone (DHT; Fig. 3K), were found in greater concentrations among males than females. In contrast to measurements using immunoassays that are more prone to cross-reactivity (9), an age-associated decline in testosterone and DHT concentrations among males was not observed, which has been reported in other studies using mass spectrometry (23, 24).

Concentrations of estrogens, including estrone (Fig. 3L) and E2 (Fig. 3M), were greater in YA females than males, with an age-associated decline occurring specifically in females. In contrast, an age-related increase in estrogens was observed in males, like other studies utilizing mass spectrometry (25). This is further demonstrated by the stronger positive correlations observed between estrone and estradiol concentrations in YA females (*r* = 0.88, *P* < 0.001) as compared to YA males (*r* = 0.60, *P* < 0.001), whereas among OA, males had a stronger correlation (*r* = 0.72, *P* < 0.001) than females (*r* = 0.65, *P* < 0.001).

### Baseline sex steroid concentrations and HA+ CD19+ B-cell responses predict seroconversion in YA more than OA

**Fig 4:**
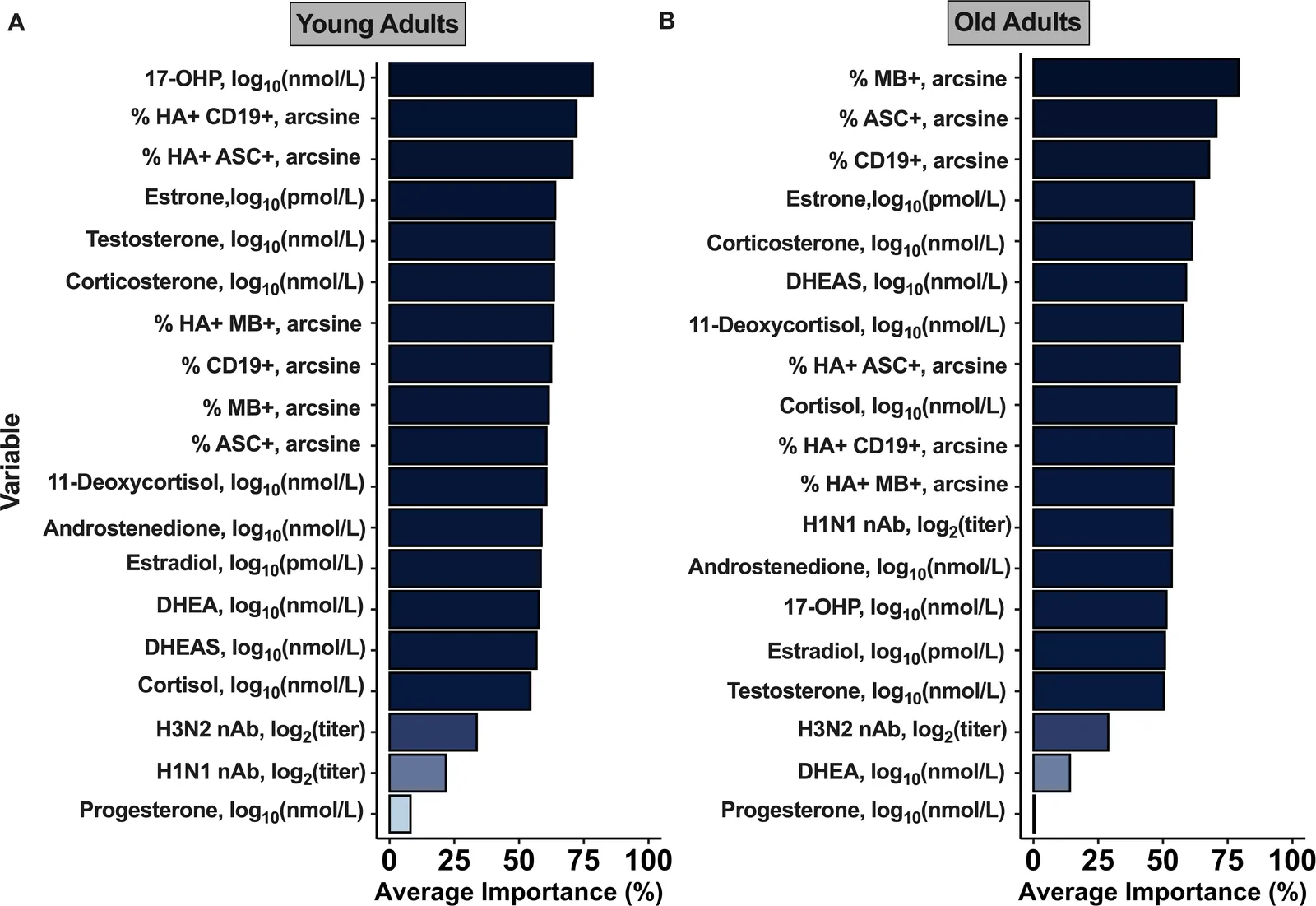
Fivefold cross-validated random forest modeling predicted seroconversion using pre-vaccination (0 DPV) measurements better among young than old adults. Data sets were downsampled and stratified by outcome variable before being partitioned, with 70% used for training the fivefold cross-validated random forest models with five repeats and averaged across 10 distinct seeds. (**A**) Variable importance plot (VIP) for the random forest model predicting seroconversion among young adults. (**B**) VIP for the random forest model predicting seroconversion among older adults. Average feature importance was calculated by averaging the mean decrease in accuracy (%) for 10 distinct seeds. Bars are colored and ranked by descending average feature importance.

To determine whether H3N2 seroconversion status could be predicted using baseline (0 DPV) measures from vaccinees, we applied a machine learning algorithm, known as random forest (RF) modeling (26), to steroid hormone, nAb, total and HA+ CD19+ B-cell subset data collected prior to vaccination for YA and OA. Complete baseline data sets without missing data were available for 27 non-responders and 44 responders among YA, while OA had 44 non-responders and 19 responders. To address potential class imbalance issues, we downsampled the data sets to the minor class, resulting in 27 responders and non-responders for YA and 19 non-responders and responders for OA (Fig. S5A and B). Using a fivefold cross-validated random forest modeling approach to predict a binary seroconversion status (responder or non-responder), which was repeated across 10 different seeds, we found that the gonadal steroid precursor, 17-OHP, % of HA+ CD19+, % of HA+ ASC, estrone, and testosterone were among the top five features most important for classifying seroconversion status for YA, as represented by the average importance (Fig. 4A). Because estrone concentrations in YA were highly correlated with estradiol concentrations prior to vaccination, particularly among YA females, estradiol may be as useful as estrone in classifying seroconversion status among YA. Averaging model performance metrics across 10 seeds, the seroconversion models for YA were able to classify the testing data with an accuracy of 60.63%, an area under the receiver operating curve (AUROC) of 0.61, and an area under the precision-recall curve (AUPRC) of 0.54 (Fig. S5A and C). In OA, the feature importance analysis following RF modeling for predicting seroconversion revealed that % MB+, % ASC+, % CD19+ B cells, and estrone were among the top features needed for classification (Fig. 4B). However, the RF models did not have strong model performance, with an average accuracy of 54%, AUROC of 0.48, and AUPRC of 0.46 (Fig. S5A and D), suggesting that baseline antibody, cellular, and steroid data are insufficient to predict subsequent seroconversion for OA.

To ensure that the RF model predictions were able to be attributed to the steroid, nAb, and cellular measures at baseline and not by random chance, we permuted the seroconversion labels of the downsampled data, performed fivefold cross-validated random forest modeling, and averaged across 10 different seeds. As expected, the RF modeling performance metrics decreased, with the average prediction accuracy of YA dropping to 50.63% and that of OA dropping to 45% (Fig. S5B, E, and F). AUROC and AUPRC also dropped to 0.48 and 0.47 for YA, respectively, and 0.38 for both metrics for OA (Fig. S5B, E, and F). These findings suggest that sex steroid concentrations and pre-existing levels of HA+ CD19+ B-cell subpopulation frequencies may be predictive of post-vaccination seroconversion status among YA, but not OA, which further underscores the age-associated role of sex steroids, including estrogens, in shaping influenza vaccine immunity.

### Age-associated differences in transcriptional activity of CD19+ B cells prior to vaccination

To explore whether the age and sex differences of nAb responses were related to the underlying transcriptional activity of CD19+ B cells, we identified a subset (Fig. S6) of YA and OA males and females with steroid concentrations, antibody responses, and CD19+ B-cell frequencies, including HA+ B-cell subsets, that were reflective of the larger cohort (Fig. 2). For the sample subset, we FACS sorted CD19+ B cells prior to single-cell RNA sequencing (scRNA-seq).

While scRNA-seq analyses comparing YA and OA participants at 7 and 28 DPV were confounded by the different doses of QIV administered as per CDC recommendations, we explored age-related transcriptional activity of B cells at “baseline” prior to vaccination. Principal component analysis (PCA) of pseudobulk gene expression revealed that prior to vaccination, the gene expression profiles of YA and OA were distinct for both males and females (Fig. S7A). Differential gene expression (DGE) analysis comparing age groups revealed that males had more differentially expressed genes (DEGs) than females (Fig. S7B). Gene set enrichment analysis (GSEA) of ranked DEGs revealed that the hallmark pathways most significantly enriched (*P*adj <0.05) in OA were tumor necrosis factor (TNF) signaling via NF-κB and inflammatory response pathways, with *SOCS3, DUSP5, NR4A3, RGSI, CD69,* and *TNSF9* being the leading genes of these pathways, for both males and females (Fig. S7C through H). These data point to an “inflammaging” of CD19+ B cells in both males and females, which has been linked to dysregulated B-cell metabolism and activity, including antibody production (27, 28). Both YA males and females had enrichment of the hallmark interferon alpha (IFNα) signaling pathway (*P*adj <0.05; Fig. S7C and D), which included a number of interferon-stimulated genes (ISGs; Fig. S7I and J) that have not been well characterized in B cells outside of production of autoantibodies (29), but may be critical in vaccine-induced antibody production.

To determine whether the age-related change in inflammatory signaling in B cells was caused by extrinsic differences in circulating cytokine and chemokine concentrations in participants, we conducted a multiplex analysis of plasma samples collected prior to vaccination. YA males and females each had greater concentrations of IL-17A than sex-matched OA (Fig. S8 and S9). OA males and females each had greater concentrations of IL-5, IL-8, CXCL10, CXCL9, and CCL5 than their YA counterparts (Fig. S8 and S9), providing some evidence of generalized inflammation among OA compared with YA, which could influence the signaling pathway differences observed in CD19+B cells with age.

### Sex differences in CD19+ B-cell transcriptional activity may be regulated by estrogens, particularly in YA

**Fig 5:**
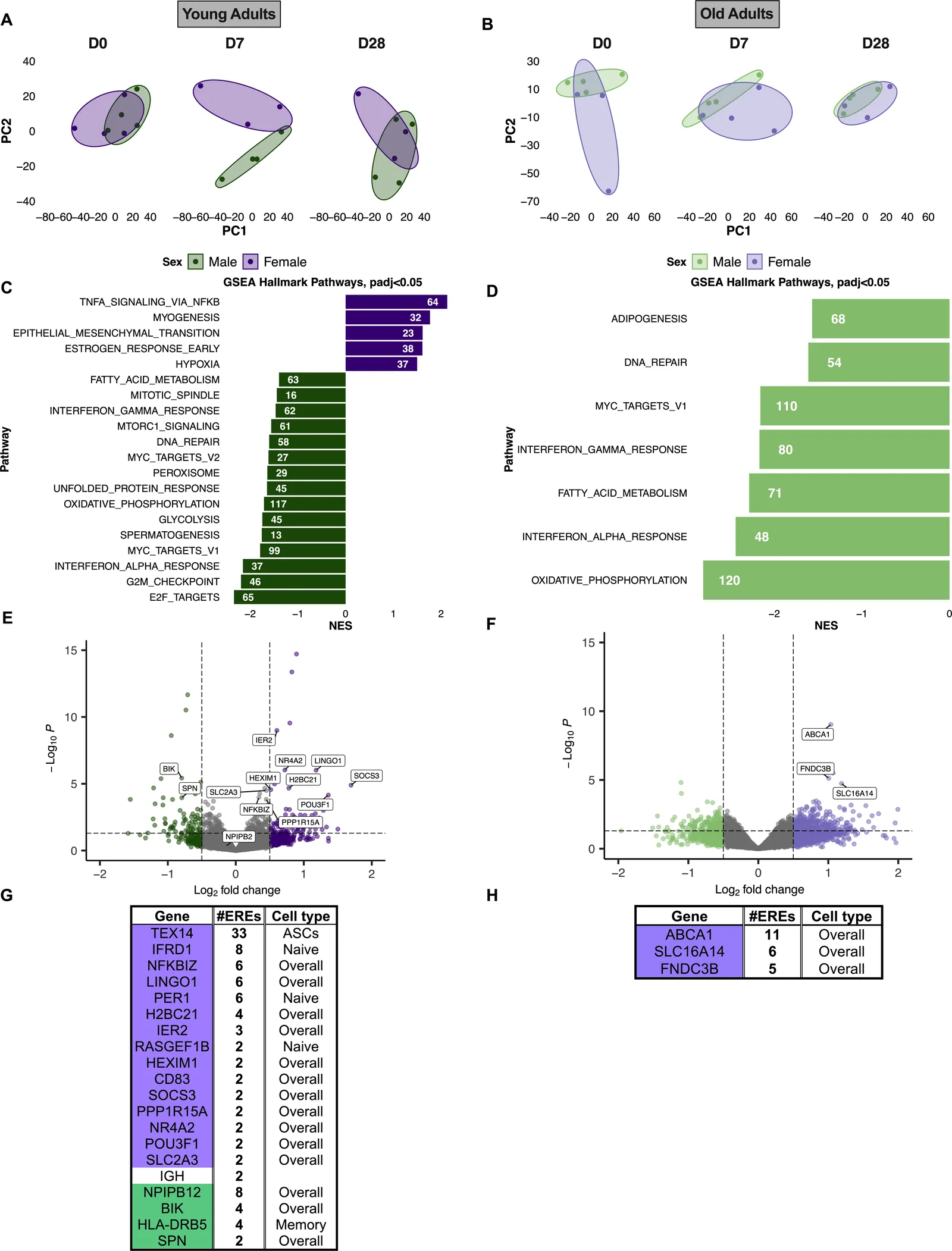
Sex differences in transcriptional activity between 7 and 0 DPV were apparent among young, but not old, adults. (**A and B**) PCA plots of pseudobulk gene expression by DPV, colored by sex (purple for females and green for males). (**C and D**) Statistically significant Hallmark gene set enrichment (GSEA) pathways (*P*adj < 0.05) are shown along with the number of leading-edge genes associated with each pathway and normalized enrichment scores (NES). (**E and F**) Volcano plots depict DEGs at *P*adj < 0.05 and log_2_FC < |0.05|, with selected DEGs for estrogen response element (ERE) analysis labeled. (**G and H**) ERE analyses were conducted using EREFinder using the top DEGs from pseudobulk analyses with ERE motifs (AGGTCAnnnTGACCT) in the promoter region (−5,000 base pairs [TSS] to +3,000 base pairs [TSS]).

To evaluate transcriptional sex differences among YA or OA, we conducted PCA of pseudobulk gene expression profiles from FACS-sorted CD19+ B cells collected at 0, 7, or 28 DPV (Fig. 5). Sex differences in pseudobulk gene expression were not observed prior to vaccination or at 28 DPV for either YA or OA. Pseudobulk gene expression patterns for males and females were distinct at 7 DPV among YA but not OA (Fig. 5A and B). At 7 DPV, CD19+ B cells from YA females had enrichment of hallmark TNF signaling via NF-κB and estrogen response signaling pathways (*P*adj <0.05), whereas CD19+ B cells of YA males had enrichment of hallmark pathways related to metabolism and IFNα signaling (*P*adj <0.05, Fig. 5C). At 7 DPV, there were no hallmark pathways enriched in OA females, but several of the same pathways related to metabolism and IFNα signaling that were enriched in YA males also were enriched in OA males (*P*adj <0.05, Fig. 5D).

To further investigate the role of estrogens in the regulation of gene expression in CD19+ B cells, we used EREFinder (30) to identify and quantify putative EREs in the promoter region (−5,000 to +3,000 base pairs) of top DEGs between males and females at 7 DPV. Among the DEGs between YA males and females at 7 DPV, we identified several genes containing EREs in their promoter regions, most of which were among the upregulated genes of YA female CD19+ B cells (Fig. 5E). Only three DEGs containing EREs were identified among OA females, with none found in the genes upregulated in the CD19+ B cells of OA males at 7 DPV (Fig. 5F). Quantification of DEGs containing EREs by EREFinder further revealed that upregulated genes in CD19+ B cells of YA females contained more EREs than those of YA males (Fig. 5G). CD19+ B cells of OA females had fewer DEGs containing EREs than YA females, with OA males having no DEGs that contained EREs (Fig. 5H). These data suggest that sex differences of CD19+ B-cell activity at 7 DPV may be due to an estrogenic regulation of gene expression via estrogen receptor binding to the consensus ERE motifs, particularly for YA females.

### Age-associated sex differences in signaling pathways in B-cell subpopulations

To define B-cell subpopulations from scRNAseq of FACS-sorted CD19+ B cells, Seurat Uniform Manifold Approximation and Projection (UMAP) clustering of single cells was used with manual cell type annotations of canonical markers (31). Apart from ABCs, which were only identified in OA, and double negative (DN) and transitional (T1-3) B cells, which were only identified in YA, ASCs, MB cells, and several populations of naïve B cells were identified in both YA and OA participants, regardless of sex (Fig. S10A through D). By proportion, naïve and MB cell populations were the greatest for YA and OA alike. Person-to-person heterogeneity was observed in the proportions of B-cell subsets, but this was not distinguishable based on the sex, age, or time point of sample collection (Fig. S10E through J).

**Fig 6:**
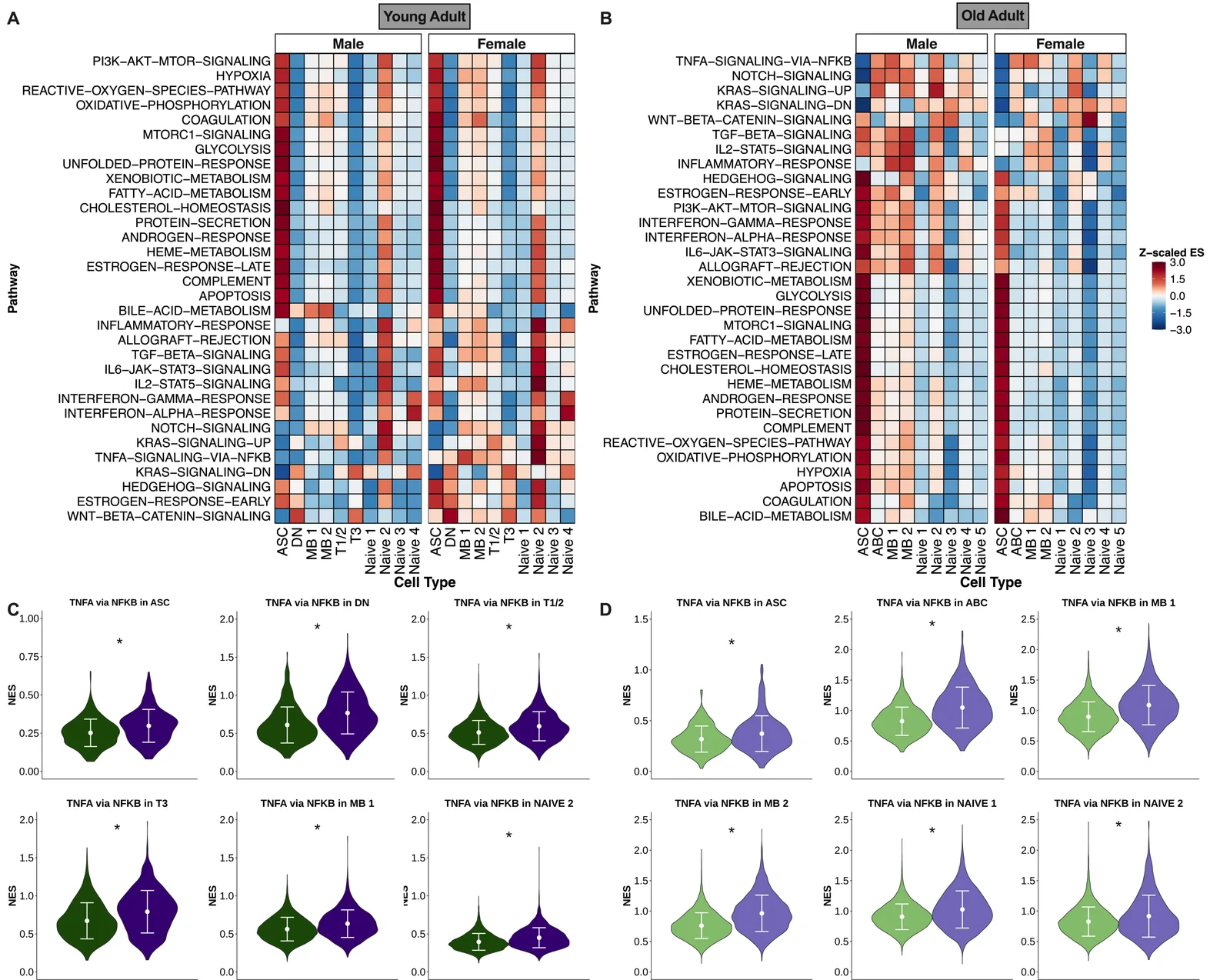
Single sample gene set enrichment analysis at 7 DPV in FACS-sorted CD19+ B cells from young and old adults. (**A and B**) Z-scaled ESs were calculated at the single-cell level using the “escape” package. Mean Z-scaled ES values are shown in the heatmaps by cell types and sex for young and old adults, respectively. Hallmark gene sets pertaining to signaling, immune, and metabolic pathways are shown. (**C and D**) Cell types with |log2FC| > 0.2 and *P*adj < 0.05 for sex differences in NES, as calculated by differential pathway analysis, with the TNF signaling via NFKB gene set being one of the most sex differential pathways in the greatest number of CD19+ B-cell subsets. *, *P* < 0.05.

Age-related sex differences were most pronounced at 7 DPV from our pseudobulk gene expression analysis; therefore, we focused our single-gene set enrichment analyses (ssGSEA) at that time point to evaluate which CD19+ B-cell subpopulations may be driving hallmark pathway enrichment in YA and OA, particularly the signaling, immune, and metabolic gene sets. Z-scaled enrichment scores (ES) were calculated for CD19+ B-cell subpopulations for each sex among YA and OA, respectively (Fig. 6A and B). For both YA and OA, we observed that ASCs, naïve, and MB cells were consistently driving the enrichment of these hallmark pathways. Inflammation and estrogen response pathways were more enriched in YA females than YA males, with the reversed pattern observed among OA (i.e., greater enrichment in OA males than females) across diverse B-cell subpopulations (Fig. 6A and B). Furthermore, the greater enrichment of the hallmark estrogen response early and TNF signaling via NF-κB pathways across several CD19+ B-cell subpopulations of YA females as compared to YA males was consistent with our pseudobulk-level findings (Fig. 5) and the greater concentrations of estrone and estradiol among YA females (Fig. 3L and M). Differential pathway analysis across CD19+ B-cell subpopulations further revealed that the hallmark TNF signaling via NF-κB pathway was consistently among the top differentially enriched pathways between males and females across most subpopulations, with greater NES in females than males, regardless of age group (*P*adj <0.05; Fig. 6C and D).

To determine whether upregulation of genes along the TNF signaling via NF-κB pathway at 7 DPV was intrinsic to CD19+ B cells from females or caused by greater overall inflammation following vaccination, plasma concentrations of cytokines and chemokines were measured at 7 DPV. Circulating cytokine and chemokine concentrations were similar between males and females among both YA and OA at 7 DPV (Fig. S11A and B). These data suggest that sex differences in the upregulation of TNF signaling via NF-κB in CD19+ B cells, particularly from YA at 7 DPV, were not caused by a sex differential inflammatory environment.

### Female cells have greater IgG secretion in response to HA antigen

**Fig 7:**
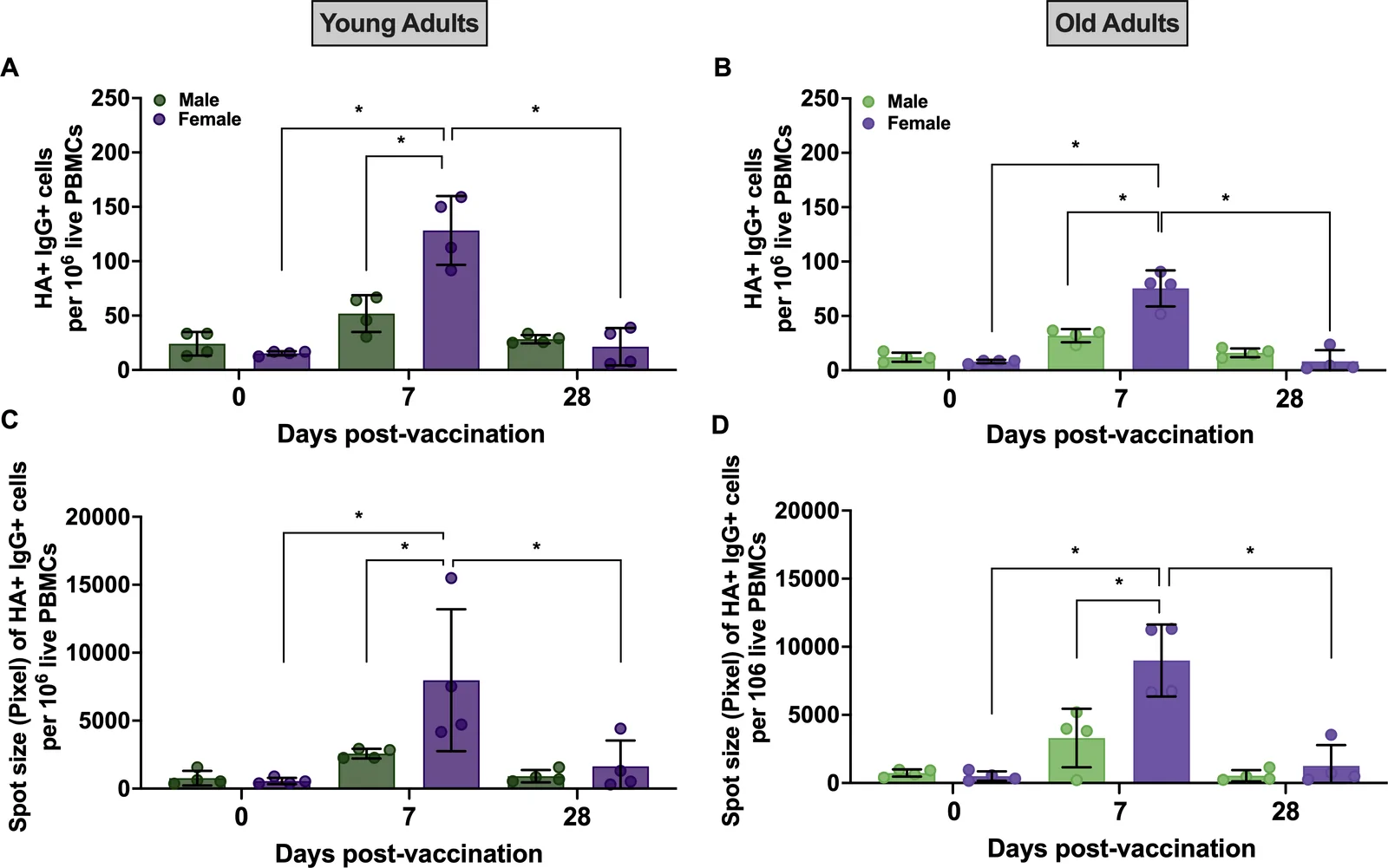
HA-specific IgG secretion is greater in PBMC samples from females than males at 7 DPV, independent of age. PBMCs from *n* = 4 males/age group and *n* = 4 females/age group participants were analyzed for HA-specific IgG+ production at 0, 7, and 28 DPV. Quantification of the number of HA-specific IgG+ cells in PBMCs from young adults (**A**) and old adults (**B**). Quantification of spot sizes (pixels) corresponding to HA-specific IgG+ in young (**C**) and old adults (**D**). Frozen PBMCs were seeded on antigen-coated plates and normalized to a total of 1 × 10⁶ PBMCs for analyses. Data were analyzed by one-way ANOVA with Tukey’s post hoc corrections. * represents *P* < 0.05.

We developed an *ex vivo* model to study the mechanisms of sex and age differences in antibody secretion following HA stimulation. We stimulated PBMCs from YA and OA females and males with H1, H3, or pooled H1/H3 antigen and analyzed HA-specific IgG production in samples collected prior to vaccination and 7 and 28 DPV. YA females, and to a lesser extent OA females, had greater numbers of HA-specific IgG+ cells than age-matched males at 7 DPV only (Fig. 7A and B). Greater numbers of HA-specific IgG+ cells were primarily in response to H3 and, to a lesser extent, H1 antigen (Fig. S12A through D). The spot sizes, corresponding to the amount of HA-specific IgG secreted per cell, in YA and OA were greater in females than males (Fig. 7C and D), which was primarily caused by greater IgG secretion in response to the H3 than H1 antigen (Fig. S12E through H). These data suggest that when cell numbers are normalized across ages and sexes, antibody secretion is greater in cells from females than males in response to HA, even among OA.

### Estrogen enhances HA+ IgG secretion via TNF and NF-κB signaling

**Fig 8:**
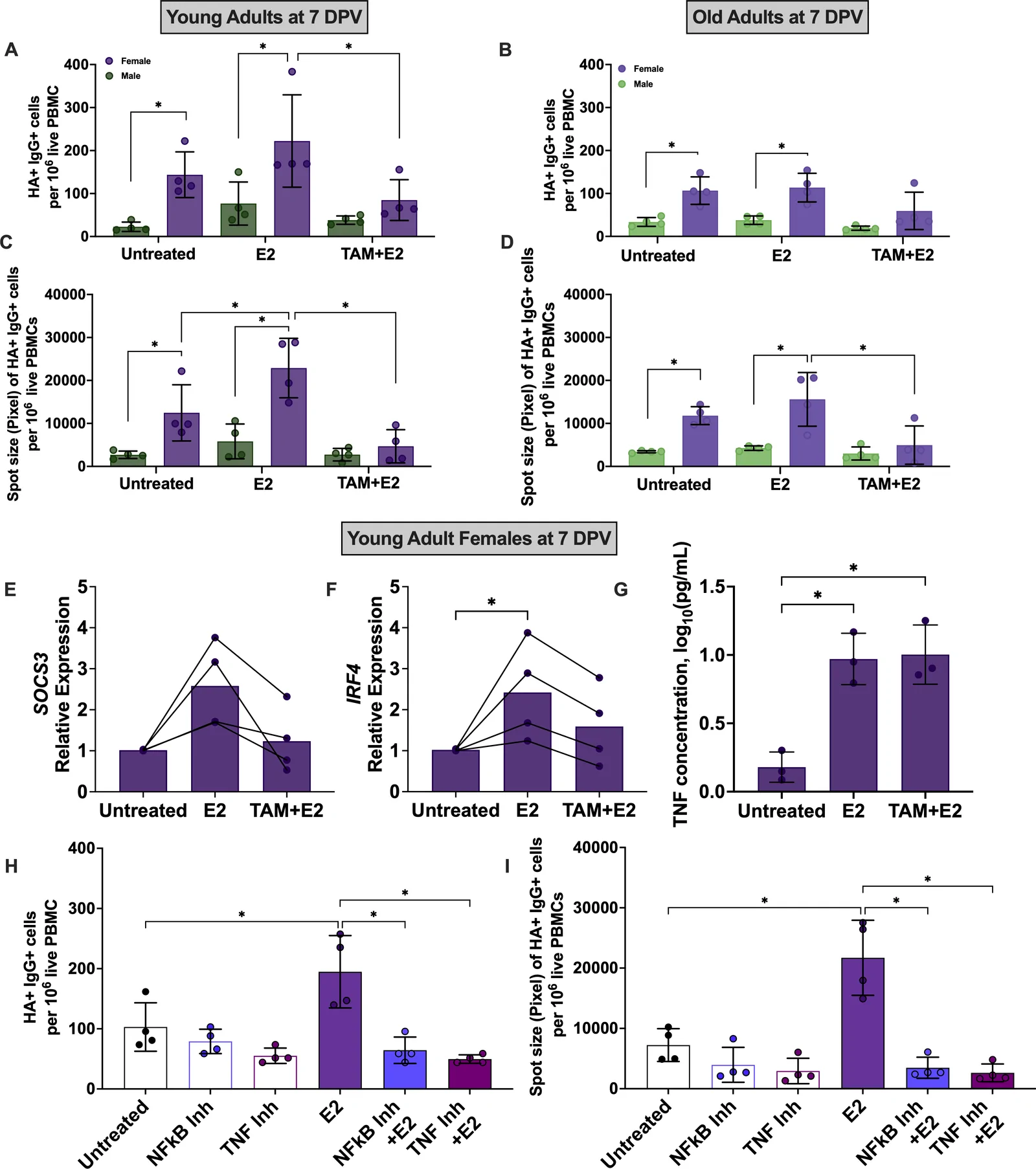
Estradiol enhances HA-specific IgG secretion through the TNF signaling via NF-κB pathway. Frozen PBMCs from YA and OA female and male participants were seeded on antigen-coated plates and normalized to a total of 1 × 10⁶ PBMCs for analyses of HA-specific IgG production at 7 DPV. Quantification of the spot numbers (**A and B**) and spot sizes (pixels; **C and D**) of HA-specific IgG+ cells 18 hours after either no treatment (untreated), estradiol (E2), or tamoxifen (TAM) followed by E2 treatment. RNA was isolated from sorted CD19+ B cells from PBMCs collected from YA females at 7 DPV and stimulated with either no treatment, E2, or TAM followed by E2 for 18 hours, and qRT-PCR was performed to measure *SOCS3* (**E**) and *IRF4* (**F**) expression. Concentrations of TNF (**G**) were measured in supernatants from PBMCs collected from the YA females at 7 DPV in panels **A** and **B**. Quantification of the number (**H**) and spot size (pixels; **I**) of HA-specific IgG+ cells 18 hours after either no treatment (untreated) or E2 treatment in the presence or absence of either 1 mM NF-κB inhibitor (Inh; Bay 11-7082) or 1 mg/mL TNF inhibitor (Etanercept). Data were analyzed by one-way ANOVA with Tukey’s post hoc corrections. * represents *P* < 0.05.

Studies in mice highlight that elevated E2 concentrations cause greater influenza vaccine-induced antibody responses in adult females than males and account for the age-related decline in immunity among females (9, 22, 32). With age-related differences in estrogen concentrations and signaling in B-cell subpopulations, including ASCs, we sought to explore whether E2 signaling through the ER in B cells caused higher antibody titers in YA females than males, which was reduced in OA. PBMCs collected at 7 DPV were stimulated with HA antigens and treated overnight with either media alone (untreated), E2, or a selective estrogen receptor modulator (SERM), tamoxifen (TAM), prior to E2. In B cells, TAM acts as an ER antagonist (33). In PBMCs from YA females, and to a lesser extent from OA females, treatment with E2 significantly increased the number (Fig. 8A and B) and size (Fig. 8C and D) of HA-specific IgG+ cells over treatment with media alone. There was no effect of E2 on cells from males. Pre-treatment with TAM before E2 significantly reduced both the number and size of HA-specific IgG+ cells in females but not males among YA and reduced the size of HA-specific IgG+ cells in OA females (Fig. 8A through D). These data illustrate that ER activation causes increased antibody secretion by B cells in females, with the cells of OA females maintaining some propensity to respond to estrogen.

Estrogen and TNF/NF-κB signaling pathways were upregulated in B cells from YA females (Fig. 5), but whether E2 increased B-cell activity via TNF/NF-κB signaling was not clear. To determine whether increased secretion of IgG in the presence of E2 was associated with increased expression of DEGs along the TNF signaling via NF-κB pathway, we sorted CD19+ B cells from PBMCs that were collected at 7 DPV from YA females and treated them with vehicle or E2 ± TAM. We used these B cells to evaluate the expression of TNF/NF-κB signaling genes, specifically *SOCS3* and *IRF4*, that were identified as having putative EREs and were upregulated in CD19+ B cells from YA females in scRNA-seq analyses. The expression of these genes was upregulated in the presence of E2 and downregulated by antagonism of ER with TAM (Fig. 8E and F). To determine whether other cells in the PBMC cultures were responding to E2 and causing upregulation of antibody secretion, TNF was measured in the supernatants of cultured PBMCs collected at 7 DPV in YA females. PBMCs stimulated with HA in the presence of E2 had greater secretion of TNF than untreated cells, which was not reversed by TAM (Fig. 8G). While TAM antagonized ER in B cells (33) , in other immune cells, including macrophages, TAM can have effects independent of ER by inducing the PI3K-NRF2 pathway to increase cytokine production (34). Because E2-induced TNF concentrations were maintained but antibody secretion was reduced by TAM in the PBMC cultures, these data suggest that the effects of ER signaling on antibody secretion in YA females were intrinsic to B cells. To determine whether E2 caused greater antibody secretion in YA females through either TNF or NF-κB signaling in B cells, we pharmacologically inhibited either NF-κB or TNF in either the presence or absence of E2. E2 could not increase either the number (Fig. 8H) or size (Fig. 8I) of HA + IgG+ cells from YA females in the absence of either NF-κB or TNF at doses that had no effect on cell viability (Table S1). These data illustrate that estrogenic enhancement of antibody secretion requires signaling through NF-κB and TNF in response to vaccine antigen.

## DISCUSSION

Sex differences in the humoral immune responses to vaccines are rarely considered and even more rarely mechanistically explored in humans. Sex differences in antibody responses are not static and can change with aging, suggesting a role for gonadal steroids. We and others have shown that females of reproductive age have greater influenza vaccine-induced nAb titers than age-matched males following receipt of either seasonal influenza vaccine H3N2 and IBV strains or novel pandemic influenza vaccines (e.g., 2009 H1N1) (9, 11, 13). In the current study, YA females had greater H3N2, but not H1N1, nAb titers and greater proportions of HA+ CD19+ B cells and HA+ memory B cells than males, which was not observed among OAs. These sex differences were primarily caused by the vaccine responders, not the non-responders who had high nAb titers prior to vaccination. These data highlight that elevated pre-existing immunity masks sex differences in humoral immunity to IAV antigens, at least among reproductive-aged individuals. It has been demonstrated that human H3N2 influenza virus evolves antigenically at a higher rate than either H1N1 or IBV (35). This translates into reduced pre-existing immunity to H3N2 in the population because antigenic site mutations in H3N2 occur and spread globally faster, leading to more frequent changes in the H3N2 vaccine component. Among OA, sex differences post-vaccination were not observed for any of the immune parameters, regardless of seroconversion status. There are different selective pressures in serum from YA versus OA recently vaccinated against influenza that result in specific amino acid changes at antigenic sites having different effects on escape from vaccine-induced immunity (36). Age-dependent antibody specificity differences might also contribute to the lack of sex differences seen in the OA, particularly if they were responding to vaccines that were stimulating strong preexisting B-cell responses, which do not show sex differences.

Antibody-secreting cells are the early producers of antigen-specific antibody that generally peak within 1 week following influenza vaccination and decline thereafter (19). Frequencies of HA+ ASCs did not exhibit a characteristic peak in the OAs but among YAs, males had greater frequencies than females at 7 DPV. When cell numbers were normalized and stimulated with HA antigen *ex vivo*, YA females, and to a lesser extent OA females, had greater numbers and sizes of HA+ IgG+ cells than their age-matched male counterparts at 7 DPV. These data highlight that the propensity for B cells to secrete IgG in response to HA antigen, particularly H3, is consistently greater for females than males, which was caused by ER signaling, as these effects were inhibited by ER antagonism. FACS-sorted CD19+ B cells from YA females also had greater transcriptional activity at 7 DPV than YA males, with upregulation of estrogen-responsive genes along NF-κB-mediated inflammatory pathways in several B-cell subsets, including ASCs and memory B cells.

Random forest models illustrated that prior to vaccination, steroids, including 17-hydroxyprogesterone, estrogens, and testosterone, as well as HA+ CD19+ B cells and ASCs, are major predictors of seroconversion to 28 DPV, particularly in YA. This provided clues for gonadal steroid mediation of greater influenza vaccine-induced immunity among reproductive-aged females as compared with age-matched males. With YA females having greater concentrations of estrogens than either YA males or OAs of either sex, we considered how estrogens could impact the activity of B cells. Estrogens have their biological effects primarily by binding to ERs in the cytoplasm of cells, including B cells, to have their canonical effects by serving as transcriptional factors that can upregulate or downregulate the expression of genes that contain EREs. In the present study, DEGs in YA females, particularly at 7 DPV, expressed more EREs in their promoters than did DEGs in either YA males or OAs of either sex. Whether ERE activation causes upregulation or downregulation of DEGs cannot be determined from our data alone. Several of the DEGs upregulated in YA females, as compared to males, however, have been empirically shown to be upregulated in the presence of estrogens in cell lines (e.g., *LINGO1, GADD45B, TEX14, POU3F1*), whereas DEGs upregulated in YA males (e.g., *BIK*) have been shown to be downregulated in the presence of estrogens (37).

In addition to directly binding EREs, cytoplasmic ERs can interact with other transcriptional factors (e.g., NF-κB) to alter gene expression, independent of EREs (38). In pseudobulk and single-cell analyses, TNF signaling via NF-κB pathways was enriched in several CD19+ B-cell subpopulations, including ASCs and memory B cells, of YA females as compared to YA males, with this sex difference being maintained in OAs. Activation of B cells with antigen can upregulate NF-κB signaling (39), which could be regulated by estrogenic activation of ERs to cause the greater magnitude of sex differences in YA than OA. NF-κB signaling is critical in the development, survival, and activity of B cells (40). TNF signaling in B cells plays roles in activation and differentiation of germinal center B cells as well as antibody production by ASCs. TNF signaling also is critical for upregulating B lymphocyte-induced maturation protein-1 in B cells to promote ASC differentiation (41), which might be greater in reproductive-aged females than males, based on our data. TNF signaling is critical for production of high-affinity antibodies to antigens (42), possibly including vaccines. Activation of IL-6 signaling pathways also was greater in YA females than males across naïve, memory, and ASCs. IL-6 activation is downstream of TNF signaling in B cells and is critical for germinal center formation, including in the context of systemic lupus erythematosus (SLE), which occurs at a ratio of 9:1 for females compared with males (43). We confirmed that estrogen and inflammatory signaling pathways interact to cause greater vaccine-induced antibody responses in YA females because E2 upregulation of antibody secretion in B cells from YA females required signaling through NF-κB and TNF. Because E2-induced secretion of TNF in PBMCs could not be reversed by TAM, but the E2-induced expression of TNF and NF-κB signaling genes and secretion of HA+ IgG in B cells was reversed by ER antagonism, these data suggest that E2 has intrinsic effects in B cells to enhance antibody secretion. While E2 has extrinsic effects on other cell types in the PBMC samples, including on the secretion of TNF, this cannot explain the intrinsic effects of ER signaling on B cells that were blocked by TAM. Because of the timing and how samples were collected, we were unable to use fresh PBMCs for ELISpot assays, which is the preferred method and affected the viability and quantity of HA+ IgG + cells in our cultures. Future studies must involve analyses of HA+ IgG+ B cells, including memory B cells, in fresh PBMCs.

Older adults manifest greater frequencies of ABCs and greater enrichment of inflammatory pathways in B cells prior to vaccination in both males and females. ABCs tend to accumulate in aged individuals and during chronic exposure to antigen, including autoantigens (44). Aging is also associated with a low-grade, systemic, and unresolved chronic inflammatory state termed inflammaging (45) or chronic low-grade inflammatory phenotype (46), with a significant impact on innate and adaptive immune responses following vaccination (47). We observed greater concentrations of inflammatory cytokines and chemokines as well as greater inflammatory profiles in OA B cells prior to the vaccine. After vaccination, aging was associated with greater IFN signaling in B-cell subsets, including ASCs and memory B cells, but only among OA males. In contrast, among YA, females trended toward having greater enrichment of IFN signaling pathways in memory B cells than males. IFN signaling, including type I IFN signaling in B cells, has been well characterized in the context of autoreactive B cells. In mice, induction of SLE results in an accumulation of ABCs with upregulation of interferon-stimulated genes and *Tlr7* gene duplication (48). Also in mice, type I IFN signaling is involved in T-cell-independent antibody production by B cells (49). Much like the pattern of estrogen concentrations, in which concentrations were greater in YA females and OA males as compared with their age-matched counterparts, it appears that IFN signaling activation, particularly in memory B cells, is elevated in these individuals after vaccination. Our previous studies in OAs that received the high-dose QIV reveal that estradiol concentrations are greater in males than females and are associated with greater concentrations of IL-6 and reports of adverse events in males compared with females (50). Sex differences and effects of aging on TNF and type I IFN pathways have been characterized in the context of developing antibody-mediated autoimmune diseases, e.g., SLE. We now show that these pathways could be important in vaccine-induced immunity, which requires further investigation.

There are several limitations to this study. We utilized two cohorts of adults, both with high levels of pre-existing immunity due to high rates of annual influenza vaccination. Our data highlight that sex differences in influenza vaccine-induced immunity are more pronounced in people with less pre-existing immunity (i.e., responders). The cohorts in the current study each received different doses of QIV, creating a confound for age-related comparisons of post-vaccine immune responses. A previous comparison between similar YA and OA cohorts in Baltimore, MD, revealed that in response to the first two doses of COVID-19 mRNA vaccine, the age-related decline in immunity was greater for males than females when OA and YA were compared (51). This study also revealed that in the absence of pre-existing immunity to SARS-CoV-2, OA females maintained greater durability of antibody over time than OA males. Together, these data further highlight that sex differences are observed in *de novo* antibody responses of OA. How steroids impact antibody responses in older individuals requires further inquiry as it appears that estrogens do not increase antibody secretion by B cells from OA males, but elevated concentrations of estrogens in OA males are associated with enrichment of estrogen response signaling pathways in B-cell subsets, like YA females. Self-reported BC use did not impact either antibody or cellular measures post-vaccination, but small sample sizes precluded deeper analysis into the type of BC used, which ranged from progesterone-only oral contraceptives and combined estrogen and progesterone oral contraceptives to non-hormonal intrauterine devices. This study did not enroll adults 50–74 years of age, a group that would encompass menopausal women. The observation that aging of cells is nonlinear, with bursts of transcriptional dysregulation occurring at ages including mid-40s and early 60s in humans (52), further highlights the need to analyze males with comparisons to females in the postmenopausal period when some females might be taking menopause hormone therapy (MHT). Future studies should consider how menopause and the use of estrogen-based MHT affect vaccine-induced immunity, as we are showing a prominent role for estrogen signaling in B cells.

Greater antibody responses among females compared with males, especially among reproductive-aged individuals, are well conserved across species and diseases (53). Whether in response to vaccine antigens, innocuous antigens (e.g., allergens), or self-antigens (e.g., autoimmunity), females mount greater antibody responses than males (2). Harnessing sex differences for public health could translate into considering different vaccine formulations based on sex, e.g., developing adjuvants to increase estrogen signaling in B cells in males of diverse adult ages and old females. This could include use of TLR7 adjuvants, as *TLR7* is both an X-linked gene that escapes X-inactivation and is more highly expressed in B cells from females than males (54, 55). *TLR7* also has EREs in its promoter (56). What if estrogens could be administered as an adjuvant with vaccines to boost B-cell signaling and antibody secretion, which could be beneficial for postmenopausal women? Our observation that *ex vivo* antigen-stimulated B cells from OA females still maintain the propensity to respond to estrogen with increased antibody secretion suggests that despite having reduced circulating concentrations of estrogens, ER signaling in B cells is still functional, regardless of aging. Prior to the U.S. Food and Drug Administration removal of the black box warning on MHT in 2025 (57), prescription and use of MHT had been underutilized (58). Since 2025, there has been a dramatic rise in the use of estrogen-based therapies in the United States (59), and studies such as this one provide mechanistic insights into how estrogens impact the functioning of diverse physiological systems, including the immune system in women.

## MATERIALS AND METHODS

### Study design

Our study involved two separate cohorts of human participants (young adults, 18–49 years old and older adults, 75+ years old). Complete sample sets were collected for 82 young adult HCWs who were enrolled through the Johns Hopkins Centers of Excellence for Influenza Research and Response (JHCEIRR) annual vaccination program. Complete sample sets were available for 84 older adults from the Johns Hopkins Longitudinal Influenza Immunization Study of Aging over 75 years of age (JH LIISA 75+). Older adults with a history of allergic reaction to influenza vaccines or to eggs, who were currently taking oral steroids or immunosuppressive medications, or who had worsening or new-onset immune-modulating conditions (e.g., rheumatoid arthritis, hematologic malignancies) were excluded. Written consent was obtained for all participants as part of the enrollment processes. Participant demographics were self-reported. Study data were collected and managed via REDCap electronic data capture tools hosted at Johns Hopkins University (60, 61). After the pre-vaccination blood draw (0 DPV), standard-dose inactivated quadrivalent influenza vaccines or high-dose inactivated quadrivalent influenza vaccines were administered intramuscularly to young adult and old adult cohorts, respectively. Blood samples were subsequently collected at 7 and 28 DPV, and PBMCs and plasma were separated and stored in liquid nitrogen or at −80°C, respectively.

### Influenza vaccine

The QIV formulation consisted of two influenza A and two influenza B strains based on WHO recommendations for the Northern Hemisphere for the 2019–2020 season: A/Brisbane/02/2018 (H1N1)pdm09-like virus, A/Kansas/14/2017 (H3N2)-like virus, B/Phuket/3073/2013-like virus (B/Yamagata/16/88 lineage), and B/Colorado/06/2017-like virus (B/Victoria-lineage/2/87 lineage) (18).

### Viruses and recombinant HA antigens

The influenza A/Brisbane/02/2018 IVR-190 virus and influenza A/Kansas/14/2017 X-327 virus were obtained from the US Centers for Disease Control and Prevention (CDC). The infectious virus titer was determined on the MDCK-Siat cell using a 50% tissue culture infectious dose (TCID50) assay as previously described (9, 62). For the flow cytometry, glycosylated recombinant HA proteins (eEnzyme) were C-terminal 6× His-tagged influenza full-length hemagglutinin (H1N1) (A/Brisbane/02/2018)-pdm09-like (amino acid 18–530) (GISAID accession# EPI1312566) or C-terminal 6× His-tagged influenza hemagglutinin (A/Kansas/14/2017/H3N2) (aa 17–529) (GenBank accession#: AXQ12067). For flow cytometry staining, recombinant HA proteins were biotinylated using the EZ-Link Micro NHS-PEG4-Biotinylation Kit (Thermo Fisher).

### Flow cytometry

Cryopreserved PBMCs were thawed and resuspended in flow washing buffer (FB), composed of phosphate-buffered saline (PBS) supplemented with 1% fetal bovine serum (FBS), 25 mM HEPES, and 1 mM EDTA. Cells were washed twice with PBS and incubated with LIVE/DEAD Fixable Aqua Dead Cell Stain dye (Thermo Fisher, #L34957) for 30 minutes at room temperature to assess viability. Live cells were identified based on exclusion of the Aqua dye. The average post-thaw viability of PBMCs was approximately 95%, with an average recovery of 80% of live cells from frozen samples. For scRNA-seq, PBMCs were stained on ice for 30 minutes with a panel of cell surface antibodies (Table S2). For HA-specific cells stimulation, PBMCs were incubated on ice for 30 minutes with 2 µg of biotinylated recombinant HA peptide pools specific to H1N1 (A/Brisbane/02/2018, aa 18–530, eEnzyme, #IA-H1-B18WP) and H3N2 (A/Kansas/14/2017, aa 17–529, eEnzyme, #IA-H3-K17WP), followed by staining with PE-conjugated streptavidin to detect biotinylated HA. HA-specific B cells were gated based on PE signal, with gating thresholds determined using a PE isotype control, PE FMO control, and PE single-color control. Staining was performed using a characterized panel of cell subtype markers (Table S2) diluted in Brilliant Staining Buffer (BD Horizon). Following staining, cells were washed twice with FB and resuspended in 200 µL of FB for acquisition on a BD Symphony A3 flow cytometer. For control conditions, unstimulated samples were processed in parallel without HA peptides, and a negative control was included using only streptavidin. Fluorescence-minus-one (FMO) controls were used for each fluorophore to assess background and determine gating thresholds. Following stimulation for flow cytometry, cells were processed for downstream analysis, including flow cytometry-based phenotyping and cell sorting. Compensation values were established before acquisition using appropriate single-stain controls. ASCs were defined as CD3e^–^CD14^–^ CD16^–^CD19^+^IgD^–^CD71^+^CD38^+^CD20^–^ cells, and MB cells were defined as CD3e^–^CD14^–^CD16^–^CD19^+^IgD^–^CD71^–^ as previously described (19) (Table S3). ABCs were defined as CD3e^–^CD14^–^CD16^–^CD19^+^CD21^–^CD11c^+^Tbet^+^, with T-bet^+^ cells identified using T-bet FMO and T-bet single-color controls (Fig. S2; Table S3). The flow cytometry results were analyzed using FlowJo v10.8 Software (BD Life Sciences).

## Data Availability

The clinical data and specimens used in this study are from the Johns Hopkins Health System (JHHS). These individual-level patient data are protected for privacy. Qualified researchers affiliated with Johns Hopkins University (JHU) may apply for access through the Johns Hopkins Institutional Review Board (IRB; https://www.hopkinsmedicine.org/institutional_review_board/). Those not affiliated with JHU seeking to collaborate may contact the corresponding author. Access to these data for research collaboration with JHU must ultimately comply with IRB and data sharing protocols. Data are available through a controlled access method that follows best practices of sharing human-subjects clinical research data at https://doi.org/10.25934/PR00017652. All code can be accessed through GitHub at https://github.com/ayin0510/Influenza-Vaccine-Human-B-Cell-Project-2019-2020.

## SUPPLEMENTAL MATERIAL

Supplemental material mbio.01435-26-s0001.pdf Additional methods, Figures S1-S12, and Tables S1-S4.

ASM does not own the copyrights to Supplemental Material that may be linked to, or accessed through, an article. The authors have granted ASM a non-exclusive, world-wide license to publish the Supplemental Material files. Please contact the corresponding author directly for reuse.
